# What is wrong with non‐respondents? Alcohol‐, drug‐ and smoking‐related mortality and morbidity in a 12‐year follow‐up study of respondents and non‐respondents in the Danish Health and Morbidity Survey

**DOI:** 10.1111/add.12939

**Published:** 2015-06-02

**Authors:** Anne Illemann Christensen, Ola Ekholm, Linsay Gray, Charlotte Glümer, Knud Juel

**Affiliations:** ^1^ National Institute of Public HealthUniversity of Southern DenmarkCopenhagenDenmark; ^2^ MRC/CSO Social and Public Health Sciences UnitUniversity of GlasgowGlasgowUK; ^3^ Department of EpidemiologyColumbia UniversityNew YorkNY; ^4^ Research Centre for Prevention and HealthThe Capital Region of DenmarkGlostrupDenmark

**Keywords:** Alcohol, bias, cause‐specific morbidity, cause‐specific mortality, drug, health survey, non‐response, smoking, survival

## Abstract

**Aim:**

Response rates in health surveys have diminished over the last two decades, making it difficult to obtain reliable information on health and health‐related risk factors in different population groups. This study compared cause‐specific mortality and morbidity among survey respondents and different types of non‐respondents to estimate alcohol‐, drug‐ and smoking‐related mortality and morbidity among non‐respondents.

**Design:**

Prospective follow‐up study of respondents and non‐respondents in two cross‐sectional health surveys.

**Setting:**

Denmark.

**Participants:**

A total sample of 39 540 Danish citizens aged 16 years or older.

**Measurements:**

Register‐based information on cause‐specific mortality and morbidity at the individual level was obtained for respondents (*n* = 28 072) and different types of non‐respondents (refusals *n* = 8954; illness/disabled *n* = 731, uncontactable *n* = 1593). Cox proportional hazards models were used to examine differences in alcohol‐, drug‐ and smoking‐related mortality and morbidity, respectively, in a 12‐year follow‐up period.

**Findings:**

Overall, non‐response was associated with a significantly increased hazard ratio (HR) of 1.56 [95% confidence interval (CI) = 1.36–1.78] for alcohol‐related morbidity, 1.88 (95% CI = 1.38–2.57) for alcohol‐related mortality, 1.55 (95% CI = 1.27–1.88) for drug‐related morbidity, 3.04 (95% CI = 1.57–5.89) for drug‐related mortality and 1.15 (95% CI = 1.03–1.29) for smoking‐related morbidity. The hazard ratio for smoking‐related mortality also tended to be higher among non‐respondents compared with respondents, although no significant association was evident (HR = 1.14; 95% CI = 0.95–1.36). Uncontactable and ill/disabled non‐respondents generally had a higher hazard ratio of alcohol‐, drug‐ and smoking‐related mortality and morbidity compared with refusal non‐respondents.

**Conclusion:**

Health survey non‐respondents in Denmark have an increased hazard ratio of alcohol‐, drug‐ and smoking‐related mortality and morbidity compared with respondents, which may indicate more unfavourable health behaviours among non‐respondents.

## Introduction

Reliable information on health and health‐related risk factors in different population groups is required to calculate the burden of morbidity and mortality attributable to these risk factors and to formulate and evaluate policies aimed at improving population health and reducing health inequalities. Health surveys of the general population are a commonly used method to obtain such information, but the validity depends upon the representativeness of the population of interest. During the last couple of decades response rates in health surveys have been diminishing, which can be problematic as it means that inference is being made on a progressively limited subsample of the population 1, 2, 3, 4. This will, however, only affect the estimates if respondents and non‐respondents differ systematically from each other in a way that is relevant to the study results. There are known differences in socio‐demographic characteristics between respondents and non‐respondents; e.g. non‐respondents are more likely to be men, young, unmarried and less educated 2, 5, 6, 7. Further, non‐respondents tend to have more unfavourable health behaviours and excess mortality than respondents 5, 6, 8, 9, 10, 11, 12. This evidence suggests that survey‐based estimates, e.g. measures of smoking and alcohol consumption, are underestimated.

Often, non‐response is assessed by comparing respondents to general population register‐based data sets 7, 13, 14, and in some cases by comparing respondents and non‐respondents on known characteristics from the sampling frame 1, 6, 10, 15. In the present study it was possible to assess mortality and morbidity among respondents and different types of non‐respondents. Previous results indicate that individuals who took the time to decline the study invitation differed from those with whom the researchers had no contact 16. Knowledge about cause‐specific mortality and morbidity among respondents and different types of non‐respondents may provide additional insight into the extent of the associated bias. This has, to our knowledge, not been assessed previously. Hence, this study seeks to expand upon and improve the evidence surrounding the well‐documented selection effects caused by non‐response.

The aim of the present study is to estimate the magnitude of non‐response bias in health surveys by comparing register‐based information on alcohol‐, drug‐ and smoking‐related mortality and morbidity, respectively, for respondents and different types of non‐respondents during a follow‐up period of up to 12 years.

## Material and Methods

The study is based on pooled data from the Danish Health and Morbidity surveys in 2000 and 2005. Both surveys are cross‐sectional and designed to be nationally representative. Information on cause‐specific death and mortality at the individual level were subsequently obtained from administrative registers and linked to all individuals in the sampling frames, including non‐respondents.

### Survey‐based data

The survey from 2000 consisted of a county‐stratified random sample of 22 484 individuals. The sample was drawn from the adult (aged 16 years or older) Danish population by the Danish National Centre for Social Research (who carried out the data collection) using the Danish Civil Registration System (each citizen has a unique personal identification number) 17. A crucial aim was to obtain at least 1000 completed interviews in each of the 15 Danish counties (except in the smallest county, where 600 completed interviews were considered sufficient). The main reason for this aim was that the survey should serve as a tool for the counties in their health‐care planning. In addition, the counties had the opportunity to increase the sample size in their county if they were willing to cover the costs that arise from such an expansion. Only one county (Frederiksborg County) decided to increase their sample size. Thus, the sample was supplemented with 612 individuals from Frederiksborg County and the total sample size was 23 096 Danish citizens. A total of 17 137 (74.2%) individuals participated in the survey. The reasons for non‐response were refusal (*n* = 5188; 22.8%), uncontactable (address of residence was obtained but the interviewer failed to establish contact to the sampled individual) (*n* = 379; 1.6%), illness/disabled (*n* = 305; 1.3%) and other reasons (e.g. linguistic barriers) (*n* = 87; 0.4%).

In 2004, a new local government reform was planned and was implemented in 2007. The counties were dissolved and five regions were established. In order to provide health‐related information to the new regions, it was decided that the survey in 2005 should obtain at least 3000 completed interviews in each region. Hence, the survey from 2005 consisted of a region‐stratified random sample of 21 832 Danish citizens, including a follow‐up sample of 5388 individuals from the 2000 survey. To avoid double counting it was decided to exclude the follow‐up sample from the 2005 sample. Thus, the final sample size in 2005 consisted of 16 444 individuals, of whom 10 935 (66.5%) participated in the survey. The reasons for non‐response were refusal (*n* = 3766; 22.9%), uncontactable (*n* = 1214; 7.4%), illness/disabled (*n* = 426; 2.6%) and other reasons (*n* = 103; 0.6%). The sample in 2005 was drawn similarly from the adult (aged 16 years or older) Danish population using the Danish Civil Registration System, and the Danish National Centre for Social Research was also responsible for the sampling and the data collection in 2005.

Institutionalized individuals were included in the sampling frame and respondents were interviewed at the institution. It is not possible to know the breakdown of reasons for non‐response for institutionalized non‐respondents from the available data material.

All selected individuals received a letter of introduction that briefly described the purpose and content of the survey, and it was emphasized that participation was voluntary. Data were collected via face‐to‐face interviews at the respondents’ places of residence (a minimum of four contact attempts) and carried out by the professional interview staff at the Danish National Centre for Social Research. More details of the survey designs are described elsewhere 1.

### Register‐based data

In Denmark, nation‐wide administrative registers are available for research purposes, and owing to the unique personal identification number it was possible to link information directly from several registers to each individual in the sample. The Danish Civil Registration System was used to retrieve information on sex, age, vital status and the date of any change of vital status. Information on the highest completed education at the time of data collection was extracted from Danish education registers, which are generated from the education institutions’ administrative records 18. Information on cause‐specific mortality was obtained from the Danish Register of Causes of Death (DRCD) 19, which covers all deaths among citizens dying in Denmark. Information on hospitalizations was obtained from the National Patient Register (DNPR), which holds administrative (e.g. hospital ward and date and time of activity) and clinical data (diagnoses and surgical procedures) on all patients in Danish hospitals 20. Classification of cause(s) of deaths and diagnoses is based on ICD‐10 codes. Table 1 displays the ICD‐10 codes used to define alcohol‐, drug‐ and smoking‐related mortality and morbidity. In‐patient admissions with one of the listed ICD‐10 codes as either a primary or secondary diagnosis were defined as events, and deaths with one of the listed ICD‐10 codes as either a primary or secondary cause were also defined as events. Only the first registered admission or death for the event under study was included in the analysis. Classifications are not mutually exclusive, which means that individuals can be classified, for example, as having both an alcohol‐related mortality event and a smoking‐related morbidity event if the individual is hospitalized with a smoking‐related event in 2008 and dies of an alcohol‐related event in 2010. The list of conditions was based mainly on a former Danish study assessing the impact on various risk factors on public health 21. All‐cause mortality was defined as any given event registered in DRCD including alcohol‐, smoking‐ and drug‐related deaths. The Danish Data Protection Agency approved the linking of the registers and the survey data and all local confidentiality and privacy requirements were met. No consent was needed at the individual level.

**Table 1:** Definitions of alcohol‐ , drug‐ and smoking‐related diagnoses.

Alcohol‐related diagnoses	Alcoholic liver disease (K70, K74)
	Acute pancreatitis (K85, K86)
	Mental and behavioural disorders due to use of alcohol (F10)
	Accidental poisoning by and exposure to alcohol (X45)
	Intentional self‐poisoning by and exposure to alcohol (X65)
	Poisoning by and exposure to alcohol, undetermined intent (Y15)
Drug‐related diagnoses	Mental and behavioural disorders due to use of drugs (F11–F19)
	Accidental poisoning by and exposure to drugs (X40–X44)
	Intentional self‐poisoning by and exposure to drugs (X60–X64)
	Poisoning by and exposure to drugs (Y10–Y14)
	Poisoning by narcotics and psychodysleptics (T40)
	Poisoning by psychotropic drugs, not elsewhere classified (T43)
Smoking‐related diagnoses	Chronic obstructive pulmonary disease (J44)
	Malignant neoplasm of larynx, tracheas, bronchus and lung (C32–C34)

### Statistical analyses

Initial descriptive analyses provided incidence rates for alcohol‐, drug‐ and smoking‐related mortality and morbidity during follow‐up and simple frequency distributions of potential confounding variables by response status. Observation intervals were calculated from the sampling date for non‐respondents (i.e. 1 January 2000 and 2005, respectively) and the interview date for respondents until the first relevant event, death, emigration or end of follow‐up (31 December 2011), whichever came first. Hence, individuals’ survival times were censored upon experiencing a competing risk; 31 December 2011 was chosen as end of follow‐up, as the longest possible follow‐up time was preferred, and 2011 was the latest year that DNPR and DRCD were fully updated at the time of data analysis. Incidence rates were calculated as the number of events during the study period divided by the sum of the person‐time of the individuals at risk. Individuals were considered at risk until the first registered admission in DNPR or death in DRCD for the event under study. The association between response status and the incidence of alcohol‐, drug‐ and smoking‐related mortality and morbidity, respectively, was analysed using the Cox proportional hazards model adjusting for potential confounding factors (survey year, sex, education). Age was applied as the underlying time in the statistical model. Evaluation of the validity of the proportional hazards assumption was performed by visual inspections of log–log plots (data not shown). The number of participants (*n*) in the models with and without the inclusion of education differs due to missing information on educational attainment. This information is missing for individuals educated abroad and the older generation. In the present data material, information on educational level is missing for 5.5% among individuals aged 16 years or older and for 45.1% among individuals aged 75 years or older. In all tests, *P*‐values were two‐sided and statistical significance was defined as *P*<0.05. All analysis was carried out using SAS version 9.3.

## Results

The relative distribution of basic characteristics among respondents and different types of non‐respondents is summarized in Table 2. In general, respondents were younger than non‐respondents and individuals with basic school education were under‐represented among the respondents. No difference was seen in the sex distribution between respondents and non‐respondents. Incidence rates of alcohol‐, smoking‐ and drug‐related mortality and morbidity were lower among respondents compared to non‐respondents; this also applied for all‐cause mortality. The associations between response status and cause‐specific mortality and morbidity, respectively, are shown in Table 3. Non‐respondents had an increased hazard ratio for alcohol‐related mortality and morbidity, respectively, compared to respondents when adjusting for survey year, sex and education. The same pattern was evident for drug‐related mortality and morbidity, smoking‐related morbidity and all‐cause mortality. Smoking‐related mortality tended to be higher among non‐respondents compared to respondents, but not significantly different. Table 4 shows the association between cause‐specific mortality and morbidity according to the different types of non‐response. Uncontactable non‐respondents had a significantly increased hazard ratio (HR) for alcohol‐related morbidity [HR = 3.17, 95% confidence interval (CI) = 2.44–4.11] and mortality (HR = 6.31, 95% CI = 3.78–10.53) compared to respondents when adjusting for survey‐year, sex and education. The same pattern was seen for refusal and ill/disabled non‐respondents, although the HRs were somewhat lower than for uncontactable non‐respondents. Further, uncontactable non‐respondents had a significantly increased hazard ratio for smoking‐related morbidity (HR = 2.25, 95% CI = 1.69–3.01) and mortality (HR = 2.79, 95% CI = 1.82–4.29) compared to respondents when adjusting for survey‐year, sex and education. The same pattern was seen for ill/disabled non‐respondents; however, the HR was somewhat lower than for uncontactable non‐respondents. No significant difference was observed between refusing non‐respondents and respondents in relation to smoking‐related morbidity or mortality. Non‐respondents who were ill/disabled had the highest hazard ratio for drug‐related morbidity (HR = 2.59, 95% CI = 1.44–4.66) and mortality (HR = 6.46, 95% CI = 1.45–28.79). Similarly, refusal and uncontactable non‐respondents had an increased hazard ratio for drug‐related morbidity and mortality, respectively. All types of non‐respondents had an increased hazard ratio for all‐cause mortality compared to respondents.

**Table 2:** Characteristics of the study population. Percentages and incidence rates (per 100 000 person‐years).

	*Respondents (n = 28 072)*	*Refusals (n = 8954)*	*Illness/disabled (n = 731)*	*Uncontactable (n = 1593)*	*P‐value*
Age (%)					
16–24 years	12.5	11.1	4.1	20.8	*P*<0.01
25–44 years	33.6	31.7	11.9	36.8	
45–64 years	34.8	36.7	19.7	26.5	
65 years or older	19.1	20.4	64.3	15.9	
Sex (%)					
Men	48.9	49.1	40.2	57.1	*P*<0.01
Women	51.1	50.9	59.8	42.9	
Educational level (%)					
Basic school	34.2	42.2	47.9	42.3	*P*<0.01
Upper secondary or vocational school	39.5	38.2	15.7	36.9	
Higher education	21.6	13.9	7.1	12.6	
No information	4.7	5.7	29.3	8.2	
All‐cause mortality [incidence rate (*n*)]	1196 (3158)	1454 (1196)	9652 (410)	2507 (276)	*P*<0.01
Alcohol‐related mortality [incidence rate (*n*)]	38 (101)	61 (50)	95 (4)	203 (22)	*P*<0.01
Alcohol‐related morbidity [incidence rate (*n*)]	218 (573)	312 (255)	547 (23)	663 (72)	*P*<0.01
Smoking‐related mortality [incidence rate (*n*)]	159 (418)	177 (145)	701 (29)	272 (30)	*P*<0.01
Smoking‐related morbidity [incidence rate (*n*)]	385 (1009)	434 (355)	1693 (70)	570 (63)	*P*<0.01
Drug‐related mortality [incidence rate (*n*)]	6 (17)	22 (18)	71 (3)	18 (2)	*P*<0.01
Drug‐related morbidity [incidence rate (*n*)]	104 (274)	158 (130)	353 (15)	291 (32)	*P*<0.01

**Table 3:** Numbers of events, hazard ratios (HR) (non‐respondents versus respondents) and 95% confidence intervals (95% CI) of all‐cause mortality and cause‐specific mortality and morbidity.

	*Adjusted for survey year*			*Adjusted for survey year, sex and education*		
	*Number of events (n = 39 540)*	*HR*	*95% CI*	*Number of events (n = 37 358)*	*HR*	*95% CI*
All‐cause mortality	5065	1.35	(1.28–1.43)	3582	1.40	(1.31–1.50)
Alcohol‐related mortality	177	2.02	(1.50–2.72)	166	1.88	(1.38–2.57)
Alcohol‐related morbidity	926	1.64	(1.43–1.87)	883	1.56	(1.36–1.78)
Smoking‐related mortality	624	1.21	(1.02–1.43)	550	1.14	(0.95–1.36)
Smoking‐related morbidity	1503	1.21	(1.08–1.34)	1360	1.15	(1.03–1.29)
Drug‐related mortality	40	3.55	(1.89–6.66)	36	3.04	(1.57–5.89)
Drug‐related morbidity	453	1.69	(1.40–2.05)	431	1.55	(1.27–1.88)

**Table 4:** Hazard ratios (HR) and 95% confidence intervals (95% CI) of all‐cause mortality and cause‐specific mortality and morbidity by response status.

	*Adjusted for survey‐year and sex*		*Adjusted for survey‐year, sex and education*	
	*HR*	*95% CI*	*HR*	*95% CI*
**All‐cause mortality**				
Respondents	1	(ref)	1	(ref)
Refusals	1.1	(1.03–1.18)	1.11	(1.03–1.20)
Illness/disabled	2.36	(2.12–2.63)	2.89	(2.51–3.34)
Uncontactable	3.46	(3.05–3.93)	3.92	(3.37–4.56)
**Alcohol‐related morbidity**				
Respondents	1	(ref)	1	(ref)
Refusals	1.40	(1.21–1.62)	1.34	(1.15–1.56)
Illness/disabled	2.52	(1.65–3.84)	2.52	(1.64–3.88)
Uncontactable	3.40	(2.65–4.38)	3.17	(2.44–4.11)
**Alcohol‐related mortality**				
Respondents	1	(ref)	1	(ref)
Refusals	1.54	(1.10–2.16)	1.52	(1.07–2.14)
Illness/disabled	2.44	(0.89–6.69)	1.94	(0.61–6.17)
Uncontactable	7.70	(4.77–12.43)	6.31	(3.78–10.53)
**Smoking‐related morbidity**				
Respondents	1	(ref)	1	(ref)
Refusals	1.04	(0.92–1.18)	1.01	(0.89–1.14)
Illness/disabled	2.06	(1.61–2.65)	2.06	(1.56–2.70)
Uncontactable	2.61	(2.02–3.39)	2.25	(1.69–3.01)
**Smoking‐related mortality**				
Respondents	1	(ref)	1	(ref)
Refusals	1.03	(0.85–1.25)	0.98	(0.80–1.20)
Illness/disabled	2.00	(1.36–2.94)	1.91	(1.22–2.98)
Uncontactable	3.21	(2.20–4.68)	2.79	(1.82–4.29)
**Drug‐related morbidity**				
Respondents	1	(ref)	1	(ref)
Refusals	1.51	(1.23–1.86)	1.41	(1.13–1.74)
Illness/disabled	3.13	(1.83–5.33)	2.59	(1.44–4.66)
Uncontactable	2.51	(1.72–3.65)	2.22	(1.52–3.26)
**Drug‐related mortality**				
Respondents	1	(ref)	1	(ref)
Refusals	3.31	(1.70–6.43)	2.86	(1.42–5.74)
Illness/disabled	8.00	(2.21–29.00)	6.46	(1.45–28.79)
Uncontactable	3.91	(0.88–17.44)	3.58	(0.80–16.12)

## Discussion

It is evident from the current study that non‐respondents were more likely than respondents to suffer from alcohol‐, smoking‐ and drug‐related morbidity and mortality. Further, the analyses showed that non‐response is a heterogeneous matter, i.e. different types of non‐response have varied effects on the non‐response bias. Refusal was the most frequent reason for non‐response, but significant differences between respondents and refusing non‐respondents was observed only in relation to alcohol‐ and drug‐related mortality and morbidity. Uncontactable non‐respondents constituted the second largest category, and pronounced differences between uncontactable non‐respondents and respondents were seen in relation to alcohol‐ and smoking‐related mortality and morbidity and drug‐related morbidity. The ill/disabled constituted a small group among non‐respondents, but had a significantly higher hazard ratio for smoking‐ and drug‐related mortality and morbidity and alcohol‐related morbidity. Overall, the observed hazard ratio was higher among uncontactable and ill/disabled non‐respondents compared to refusal non‐respondents. Hence, uncontactable and ill/disable non‐respondents were the most important contributors to non‐response bias.

The assumption in the present study is that differences in alcohol‐, smoking‐ and drug‐related morbidity and mortality infer differences in alcohol consumption, smoking and drug use. However, this requires a strong association between the selected ICD‐10 codes and the particular health behaviour. Smoking is a fairly specific cause for chronic obstructive pulmonary disease and cancer in the larynx, tracheas, bronchus and lung, which were the diagnoses selected to represent smoking. Overall, the aetiological fraction of smoking in the development of these diseases is approximately 80–90% 22. The aetiological fraction for each of the selected alcohol‐ and drug‐related diagnoses is 100% per definition. The chosen alcohol‐ and drug‐related diseases are, however, associated mainly with extensive alcohol consumption and drug use, respectively, and diseases associated with more moderate use are not included. The results, therefore, indicate more heavy alcohol and drug use and not necessarily more moderate use among non‐respondents than respondents. Thus the selected diagnoses provide guideline indications of the actual consumption in the general population and the results indicate that estimates of smoking and heavy alcohol and drug use are most probably underestimated by the bias produced by non‐response. This is in accordance with previous studies 6, 9, 10, 15, 23, 24. Such resultant bias is an important component that should be taken into consideration in surveys based on general populations 14.

The reason why smoking‐related events show less relationship with non‐response compared to alcohol‐ and drug‐related events may be explained partly as follows: as described, the alcohol‐ and drug‐related diagnoses are associated with extensive alcohol consumption and drug use, and individuals with these diagnoses may represent a more marginalized group with more severe health problems than heavy smokers. Further, the selected alcohol‐ and drug‐related diagnoses have a shorter lag time than the selected smoking‐related diagnoses. Hence, non‐respondents may have died from other causes before developing a smoking‐related diagnosis.

Some degree of health outcome‐related self‐selection into the study is to be expected; e.g. those who are already sick at baseline decline to respond resulting in a higher morbidity and mortality among non‐respondents during the first years of follow‐up. This is supported by a previous study, showing that that the excess mortality of non‐respondents was higher after 4 years of follow‐up in comparison to 28 years of follow‐up 10. However, a stable excess mortality and morbidity several years after baseline would indicate that respondents and non‐respondents differ not only in health status at baseline. The analysis in the present study showed differences in mortality and morbidity between respondents and non‐respondents even after a relatively long follow‐up period, and differences in life‐style are therefore likely. Differences in socio‐demographic characteristics between respondents and non‐respondents have been suggested as an explanation for this difference in outcome, but this factor did not seem to be a sufficient explanation in the current study. In most cases the adjusted HR remained significantly increased for non‐respondents, indicating that these socio‐demographic characteristics did not capture all the differences in health between the groups. This is in accordance with findings in other studies 10, 23. One explanation could be that sex and education captured inadequately the factors related to participation, i.e. the categories were too broad or that other unmeasured characteristics were more important. Another explanation could be that those who chose to respond were healthier than those who chose not to respond, even within the same socio‐demographic group.

As mentioned in the Statistics section, information on educational level is missing for individuals educated abroad and the older generation. However, as this is a problem for both respondents and non‐respondents there is no reason to suspect any differential misclassification of educational level.

When using DNRP there are some aspects one needs to be aware of. Since 2000 the DNPR has formed the basis for payment to public hospitals, and the registration from these hospitals is assumed to be complete from that time. However, registration from private hospitals and clinics is known to be incomplete. In 2008, the National Board of Health estimated that 5% of all operations were missing from the DNPR. In addition, hospitals in Denmark are reimbursed according to the diagnosis‐related groups system (DRG), which is a classification system that identified the ‘products’ that the patient has received. Hence, the use of the DRG system for payment of hospitals may cause a diagnostic drift in the coding towards diagnoses with higher costs, which influences the validity of disease classifications in hospital systems 25. However, any misclassification of outcome will most probably be non‐differential, as it will apply for both respondents and non‐respondents, and will therefore tend to underestimate the true association. Lastly, DNPR and DRCD only cover morbidity and mortality events that occur in Denmark. Hence, individuals who emigrate have unknown health outcomes and are therefore censored from the analysis at the day of emigration.

Overall, the identified non‐response bias is an important component that should be taken into consideration when using estimates based on surveys of the general population. Standard approaches aim to improve overall response rates, but even if the response rate is high selective non‐response may bias estimates, and tailored methods aiming to improve response rate in different types of non‐response groups—particularly hard‐to‐reach communities and those with morbidities—may be warranted. For example, the introduction letter could stress the importance of response despite illness/disability, and a shorter questionnaire could be offered as a second option to those indicating illness/disability. Additionally, the number of contacts attempts could be increased for uncountable non‐respondents and the contact attempts could be varied by time of day and week and by contact mode, i.e. telephone, letter and visit to the address.

*Post‐hoc*, non‐response adjustment by inverse probability weighting is applied routinely to account for non‐response bias by selective non‐response. However, weights based on socio‐demographics characteristics may not capture adequately differential health and health‐related behaviour within categories. The information obtained from the present study may be used to improve weighting, as the success of weighting depends upon how useful the proxy variables (reason for non‐response) are for the survey variables (health behaviour). This will never substitute fully for missing data from non‐respondents, but knowledge concerning socio‐demographic characteristics and cause‐specific mortality and morbidity among respondents and different types of non‐respondents may provide additional insight into the magnitude and direction of the associated bias. However, this requires that information on reason for non‐response be registered in a systematic way during data collection. A promising alternative to weighting is a health outcome‐informed multiple imputation approach, which is currently in development 26. This exploits the record‐linked health outcome data to form the basis of imputation of missing survey data on non‐respondents. The explicit incorporation of differential distributions for respondents and different kinds of non‐respondents can be factored in by implementation of a pattern mixture‐based approach which allows for data which are missing not at random 27.

No further information, such as diagnosis, is available about illness or disability from the present data material. This information would have been valuable to further explore this reason for non‐response, but unfortunately it was not registered during data collection.

## Conclusion

The increased hazard ratios of alcohol‐, drug‐ and smoking‐related mortality and morbidity among non‐respondents compared to respondents indicate more unfavourable health behaviours among non‐respondents compared to non‐respondents. Further, different types of non‐response seemed to have varied effects on non‐response bias. To reduce the selection bias, data collection strategies that maximize the response rate among those non‐respondents who are the most important contributors to non‐response bias should be used, and *post‐hoc* methodologies such as tailored multiple imputation using information on reasons for non‐response could be applied.

### Ethical considerations

The Danish Data Protection Agency has approved the linking of the registers and the survey data, and all local confidentiality and privacy requirements have been met.

### Declaration of interests

None.

### Acknowledgement

The UK Medical Research Council and the Chief Scientist Office of the Scottish Government Health Directorates (CSO) funds Linsay Gray as part of the Measuring Health programme [MC_UU_12017/5 and SPHSU2] at the MRC/CSO Social and Public Health Sciences Unit, University of Glasgow; Linsay Gray is also funded by the CSO [MC_A540_ 5TK10].
